# Effective Methods of Estimation of Pathogen Prevalence in Pooled Ticks

**DOI:** 10.3390/pathogens12040557

**Published:** 2023-04-05

**Authors:** Gerardo Fracasso, Marika Grillini, Laura Grassi, Francesco Gradoni, Graziana da Rold, Michela Bertola

**Affiliations:** 1 Eco-Epidemiology Group, Department of Biomedical Sciences, Institute of Tropical Medicine, 2000 Antwerp, Belgium; 2 Evolutionary Ecology Group, Department of Biology, University of Antwerp, 2610 Wilrijk, Belgium; 3 Department of Animal Medicine, Production and Health, University of Padua, 35020 Legnaro, Italy; 4 Istituto Zooprofilattico Sperimentale delle Venezie, 35020 Legnaro, Italy

**Keywords:** tick-borne diseases, tick-borne pathogens, pool positivity rate, minimum infection rate, pooled prevalence, PPR, MIR, EPP, pooling

## Abstract

Since tick-borne diseases (TBDs) incidence, both in human and animal populations, is increasing worldwide, there is the need to assess the presence, distribution and prevalence of tick-borne pathogens. Reliable estimates on tick-borne pathogens (TBPs) prevalence represent the public health foundation to create risk maps and take effective prevention and control actions against TBDs. Tick surveillance consists of collecting and testing (usually in pools) thousands of specimens. Construction and analysis of tick pools represent a challenge due to the complexity of tick-borne pathogens and tick-borne diseases ecology. The aim of this study is to provide a practical guideline on appropriate pooling strategies and statistical analysis of infection prevalence through: (i) reporting the different pooling strategies and statistical methodologies commonly used to calculate pathogen prevalence in tick populations and (ii) practical comparison between statistical methods utilising a real dataset of infection prevalence in ticks collected in Northern Italy. Reporting detailed information on tick pool composition and size is as important as the correct TBPs prevalence estimation. Among the prevalence indexes, we suggest using maximum-likelihood estimates of pooled prevalence instead of minimum infection rate or pool positivity rate given the merits of the method and availability of software.

## 1. Introduction

Targeted surveillance for pathogens within arthropod vector populations offers the ability to detect pathogens (i.e., bacteria, viruses and protozoa) prior to their (re)emergence in animal or human populations. This establishes a reference baseline of vector-borne pathogen activity and allows to monitor its change over time.

During the last decades, ticks increased in density and geographical range (both to higher altitudes and latitudes). This pattern is expected to exacerbate with climate change. In fact, predicted temperature increases could cause tick range expansions, prolonged tick activity, changes to tick development rate, reproduction and survival, as well as changes to the development rates of the pathogens these vectors may carry [1,2,3]. In light of the above, it becomes extremely important to pay attention to tick surveillance and tick-borne pathogens (TBPs) screening. Effective tick-based surveillance is, in fact, essential for monitoring human and animal disease emergence and spread. Data collected during surveillance activities are pivotal to implement adequate prevention and control measures, as they provide information about the risk of exposure in human and animal populations and the spread and distribution of both vectors and pathogens [4].

Often, arthropod vectors are collected in large numbers during a single sampling activity, thus requiring long processing times. Additionally, due to the high costs of diagnostics, it may not be possible to investigate specimens individually and evaluate their individual-based pathogen prevalence. For these logistic and economic reasons, pool screening represents a method conventionally used to analyse arthropod vectors in veterinary and human medicine since 1943 [5,6,7,8,9].

Among vectors, tick pools represent a challenge due to the complexity of TBPs and tick-borne disease (TBD) epidemiology (e.g., densities of vectors and hosts, environmental and climatic variables). Indeed, pool prevalence analyses should take into account that TBPs are associated with the development, survival and reproductive rate of ticks [10] and to the presence and densities of reservoir-competent hosts. Moreover, climatic and microclimatic conditions (such as temperature and relative humidity) and environmental parameters affect tick activity and behaviour and, consequently, the transmission of TBPs [11].

Differently from other pathogens, which generally occur at very low prevalence (<0.01%) [12], it is not uncommon for TBPs to reach a prevalence of up to 40% [13,14]. Importantly, the stage of tick development significantly affects TBP prevalence and transmission. In fact, depending on the pathogen species, there might be transstadial (i.e., a pathogen is transmitted throughout the moult), vertical (from the female to its eggs) and horizontal transmission (i.e., during co-feeding on the host), respectively [15,16]. 

Usually, tick surveillance consists of collecting and testing thousands of specimens, and it is thus more practical and cost-effective to pool samples according to different criteria such as life stage or sex. In the literature, different methods to pool ticks are reported and mainly split specimens into pools of variable size [17,18,19] or fixed size [20,21,22,23]. At this stage, it should be pointed out that an incorrect pooling strategy can lead to a severely over- or underestimated infection prevalence for several reasons. First, in a pool that tested positive for a pathogen, the exact number of infected specimens in the pool itself cannot be determined [24]. Second, diagnostic methods might incur a reduction in sensitivity (detection of true positive samples) for pools with a high number of specimens and low infection prevalence or high engorgement status (dilution effect). Third, pools might be spatially or temporally inhomogeneous between each other, e.g., with some pools made of ticks from the same area (or day). Fourth, pools might contain different life stages with different likelihoods of infection. The latter, besides giving biased infection prevalence estimates, also makes comparisons between life stages impossible. 

Besides the pooling strategy, statistical methods to estimate infection prevalence could also add biases, and it is crucial for the researcher to understand their caveats in order to appropriately use them. In the literature, three main calculation methods are used: (i) pool positivity rate (PPR), (ii) minimum infection rate (MIR) and (iii) maximum-likelihood estimate of pooled prevalence (EPP). 

The pool positivity rate (PPR) is calculated as the ratio of the number of positive pools to the total of pools tested [13]. 

Minimum infection rate (MIR), which is calculated as the ratio of the number of positive pools to the total number of specimens tested [8,25], is the most basic and widely used method for the analyses of pooled samples. However, despite its extensive use, it is strongly influenced by the pool size, which is chosen arbitrarily. The underlying assumption of the MIR is that only one infected individual exists in a positive pool, and for this reason, it estimates the lower bound of the infection rate [8,26,27]. As a matter of fact, MIR will often underestimate the actual prevalence as positive pools will be increasingly likely to include more than one positive tick at the increase of the pool size and true pathogen prevalence. 

The maximum-likelihood estimate of pooled prevalence (EPP) is defined as the infection rate most likely observed given the test results and an assumed probabilistic model (i.e., a binomial distribution of infected individuals in a positive pool). This method could be set both with fixed or variable pool size and, assuming perfect 100% test sensitivity and specificity, permits to estimate prevalence and confidence limits [28,29].

The present study aims, firstly, through a comprehensive literature screening, to investigate and compare the main pooling strategies and statistical methodologies used to calculate TBPs prevalence in tick populations. Secondly, we discuss the correct use of such pooling strategies and statistical methodologies. Finally, a real dataset of infection prevalence in ticks collected in Northeastern Italy is analysed to provide a practical comparison between statistical methods.

## 2. Materials and Methods

### 2.1. Search Strategy, Study Selection and Data Analysis

We performed a literature review using the PubMed online database with the following keywords: “infection rate”, “positivity rate”, “prevalence”, “tick-borne pathogen” and “pool”. This search includes all pooled prevalence studies (full-text) found using PubMed up to 31st December 2022. Grey literature (e.g., theses, conference presentations and abstracts) were not included. An eligible study was defined as one that collected original data on TBPs from either pooled or individual ticks.

A database including each selected article was created reporting the following information: bibliographic details (title, authors, journal, publication year, doi); pool details, namely individual or pooled ticks, fixed or variable pool and pool size; tick stadium (adult, nymph, larva); prevalence indexes used, such as the number of positive pools, infection rate, minimum infection rate, maximum infection rate, pool positivity rate and estimated pooled prevalence; if confidential intervals were calculated; software or package used during the data elaboration and relative references.

### 2.2. Tested Dataset and Statistical Analysis

The ticks and TBPs dataset was retrieved from Bertola et al. [13] and consists of 2668 questing *Ixodes ricinus* (all stages) collected by dragging in 20 municipalities in a highly endemic area for TBPs in Northeastern Italy (Belluno province, Veneto region) each year (from April to November) from 2011 to 2017. Collected ticks were pooled according to their life stage, sex, date and sampling site resulting in a total of 596 pools. Single adult ticks, pooled nymphs (maximum 13 specimens per pool) and larvae (maximum 22 specimens per pool) were screened for nine different TBPs circulating in the area. For this dataset, we calculated the PPR, MIR and EPP considering positivity to (i) any of the nine TBPs screened, (ii) the TBP with lowest prevalence, i.e., *Borrelia garinii* and (iii) the TBP with highest prevalence, i.e., *Rickettsia helvetica*. For each of these three scenarios, estimates were computed considering (a) all pools including different pool sizes (variable pool size), (b) only pools made of two individuals (fixed, small pool size) and (c) only pools of ten individuals (fixed, high pool size). Analyses were carried out in R software, version 4.2.2 [30]. EPP estimates were calculated using the “PoolTestR” package [31].

## 3. Results

### 3.1. Summary of Literature Data

For this study, 235 documents were retrieved from the Pubmed database and 191 papers were considered eligible for inclusion. From the retrieved literature, TBPs screening in ticks was performed through pool screening of all the specimens collected (65%, 124/191) or mixed individual and pool screening (32%, 61/191). Few papers (6/191) analysed the specimens collected only at the individual level. Specifically, individual screening was mostly based on the developmental stage, although other parameters were also chosen (e.g., tick species, sex, engorged status, animal host and locality).

Among the 185 studies in which ticks have been analysed in pools, 144 used a variable pool size (from 1 to 70 specimens), 34 a fixed pool size (from 1 to 40 specimens) and 7 studies used both methods.

Often there was no indication on the developmental stage of analysed ticks (*n* = 21) or sex in case of adult specimens (*n* = 53). Crucial methodological information such as the pool size was missing in 35 papers.

Different developmental stages were often analysed with different approaches (e.g., adults individually and nymphs and larvae in pools), but in 30 out of 96 papers (31%), more than one stage was analysed by pooling together different stages.

As expected, three main methods were used to estimate TBP prevalence in pooled ticks: PPR, MIR and EPP (Table 1).

### 3.2. Tested Dataset and Statistical Analysis

**Figure 1:**
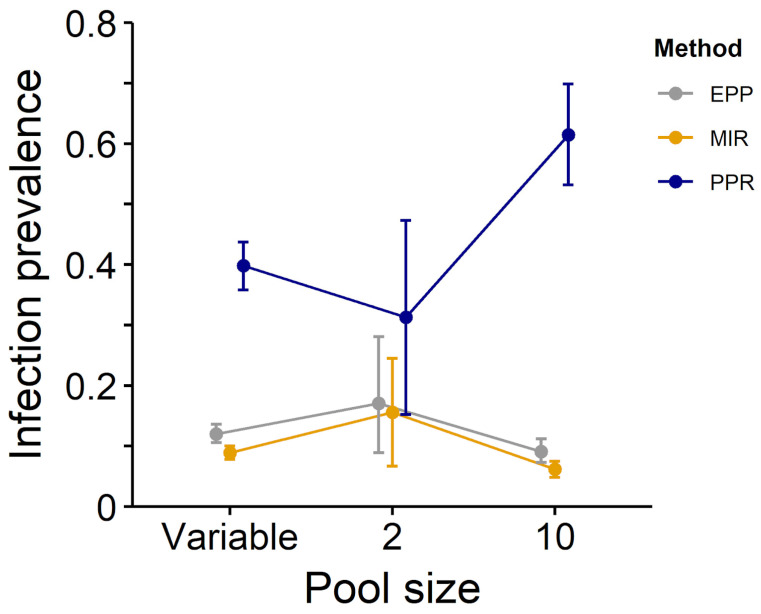
Estimates of infection prevalence for at least one of nine tick-borne pathogens using the PPR (blue), MIR (yellow) and EPP (grey) methods (dataset composed by ticks collected in Northeastern Italy from 2011 to 2017).

**Figure 2:**
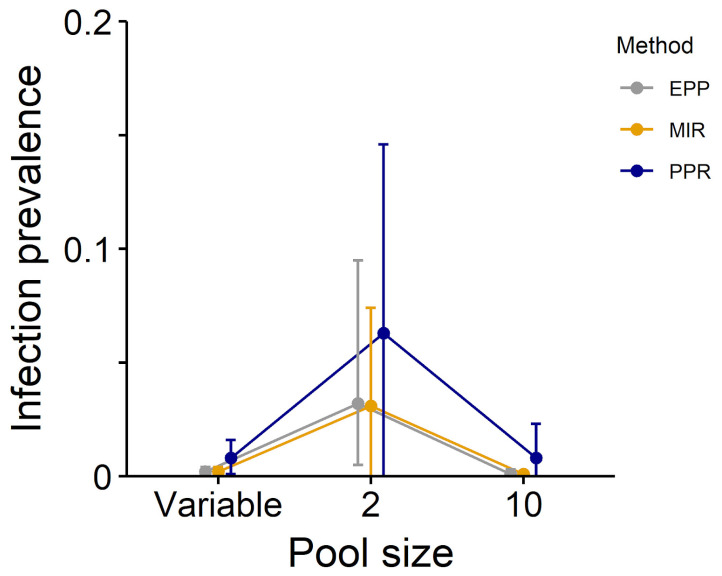
Estimates of infection prevalence for a tick-borne pathogen with low prevalence in the study area (*Borrelia garinii/afzelii*) using the PPR (blue), MIR (yellow) and EPP (grey) methods (dataset composed by ticks collected in Northeastern Italy from 2011 to 2017).

**Figure 3:**
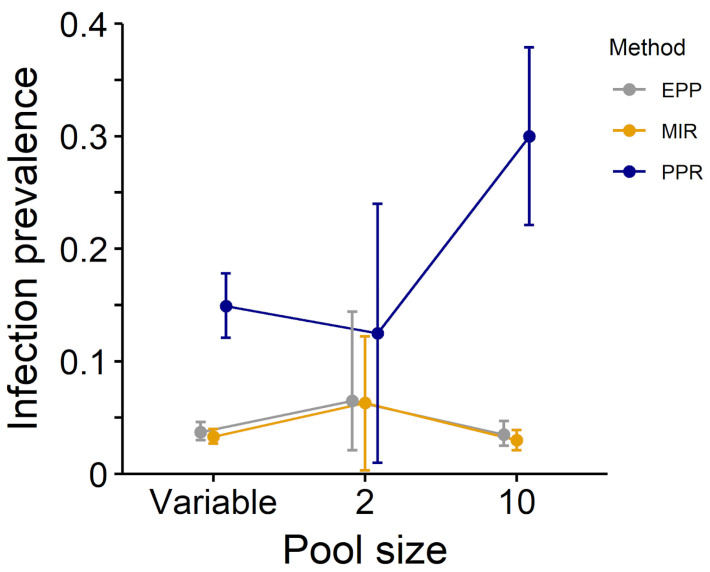
Estimates of infection prevalence for a tick-borne pathogen with relatively high prevalence in the study area (*Rickettsia helvetica*) using the PPR (blue), MIR (yellow) and EPP (grey) methods (dataset composed by ticks collected in Northeastern Italy from 2011 to 2017).

When comparing these three methods with our real-world dataset, we obtained similar estimates between EPP and MIR estimates, while PPR estimates differed significantly in several instances (Table 2, Figure 1, Figure 2 and Figure 3).

## 4. Discussion

Pooled analysis permits to optimise resources and obtain an estimated prevalence measure. After tick collection and identification, the most common pooling method for TBPs screening is the creation of pools with variable size based mainly on the sampling site or host species. The use of a variable pool size has been shown to provide more accurate estimates of infection prevalence and should be preferred.

Given our findings and the available literature on the topic, we would like to highlight the following key points.

In case of known presence of TBPs in a certain area, a sample of ticks and trials with different pool sizes should be performed in advance to estimate the proper amount of specimens required to accurately estimate the TBPs prevalence [28,42,43].

Punctual and precise description of the pooled specimens (stage, sex, engorged status and pool size) is needed to easily compare results from different geographical areas, time of the year and collection methods during the statistical analysis. It is of pivotal importance to never mix different life stages in a single pool. In this way, ecological, physiological and life-history differences between stages will not confound estimates of infection prevalence. Additionally, results can thus be readily compared between studies.

As in the case of tick-borne encephalitis studies, uninfected ticks from the surrounding areas of an endemic foci should not be included in the analysed specimens to avoid a prevalence underestimation [44]. Overall, it is crucial to always keep in mind the ecology of the TBPs investigated as well as that of their vectors as many biases can be tackled at the stage of study design and data analysis.

In addition, different methods for detecting TBPs can have different sensitivities. For this reason, it is important, in case of low TBPs concentration or undetectable state in ticks (i.e., non-active TBPs replication, not recently fed ticks or pools with large proportion of immature stages compared to adults), to analyse small size pools in order to not underestimate the infection prevalence.

When choosing the maximum pool size, care should be taken to avoid dilution effects and thus a reduction in sensitivity (detection of true positive samples). Tests with known positive and negative samples should be carried out to determine the optimal pool size based on pathogen detection sensitivity, workload and costs.

An appropriate statistical method should be adopted according to the ecological and epidemiological framework. Out of the three main methods used for estimating infection rates from pooled samples, considering the results herein reported, the use of EPP should be preferred over other methods due to its precision and flexibility in accounting for spatio-temporal confounding effects [31].Overall, the EPP is more accurate and robust than the MIR and requires no more data than those used to calculate the MIR itself [45].Of note, the use of statistical tools such as PoolTestR also give the important advantage of accounting for covariates and hierarchical sampling, thus providing estimates that are more accurate. For hierarchical sampling, we mean sites nested within one or more levels, e.g., sampling sites within towns and towns within regions. Although EPP has been presented several decades ago [46], this method has not been widely appreciated and applied by medical entomologists. We believe that EPP may replace MIR in measuring infection rates given the merits of the method and software availability.

It has been suggested that the MIR index should be effectively used mainly in case of very low TBPs prevalence (e.g., <0.1%) [8]. Our results show that MIR and EPP provided similar estimates in different scenarios and with different pooling strategies using real infection prevalence data. Although the values generated by the two methods are not typically different when infection rates are low and pool sizes small, the difference can be significant when these conditions are violated. In general, we suggest to calculate both the EPP and MIR estimates whenever possible to allow the comparison between studies using different techniques and separated in time and space. Additionally, we noticed that prevalence obtained from pooled samples with MIR and EPP methods is often reported without confidence intervals (CI); such results, if interpreted as from a single individual, can overestimate the true prevalence in the population [41]. For this reason, MIR and EPP should always be associated with CI.

Lastly, although PPR is the prevalence index most frequently used, it is a “gross” index, highly dependent on pool size and infection rate of ticks.

## 5. Conclusions

In the near future, tick surveillance and TBPs screening are expected to become public health routine activities. Pooled analysis of the collected specimens permits to optimise resources and obtain a reliable estimated prevalence only if sampling and pooling strategies are correctly applied. Solid estimates of TBPs prevalence are the foundation to create risk maps and take prevention and control actions against TBDs.

We suggest future studies to carry out preliminary tests to determine a sensible pooling strategy, i.e., the optimal trade-off between test sensitivity, workload and costs. Furthermore, we suggest to use a variable pool size and prefer the EPP method over the others for its reliability and flexibility.

## Figures and Tables

**Table 1:** Prevalence indexes (i.e., PPR, MIR and EPP) used to analyse tick pools reported in the retrieved literature.

Prevalence Index	Papers Using the Method/Total Papers	Associated with Confidence Interval (CI) 95%	Specific Methods/Software/Package	Association between Different Indexes
Pool positivity rate (PPR)	106/185 * (57%)	-	-	PPR could be associated with MIR (*n* = 17)
Minimum infection rate (MIR)	64/185 ** (35%)	18/184 (10%)	PooledInfRate software in Excel [32] Epitools^®^ calculator [33] Epi Info program ver. 6 [34] Quantitative Parasitology 3.0 (QP 3.0) software [35,36]	MIR could be associated (*n* = 4) with maximum infection rate (MaxIR) index, which was calculated by considering that all the ticks present in a positive pool are infected.
Estimate pool prevalence (EPP)	35/185 (19%)	26/35 (74%)	PooledInfRate software in Excel [32] Method of Hauck [37] Method of Krebs [38] Method of Cowling [8] R package by Devleesschauwer [39] R package by McLure [31] Method of McV [40] Williams & Moffitt [41]	EPP could be associated with MIR (*n* = 4) or PPR (*n* = 1)

* Only 43 papers properly used the PPR definition, the remaining papers simply resume TBPs pool positivity, reporting the number of positive pools obtained without dividing by the number of total pools screened. ** In one paper, MIR has been calculated improperly as: 1/N specimen in the largest pool and MaxIR as: 1/N specimen in the smallest pool.

**Table 2:** Prevalence indexes and confidence intervals (CI) considering different level of pool positivity and sizes (dataset composed by ticks collected in Northeastern Italy from 2011 to 2017).

Dataset	PPR (CI)	MIR (CI)	EPP (CI)
All diseases, unequal pools	0.398 (0.358–0.437) *	0.089 (0.078–0.100) *	0.120 (0.106–0.136)
All diseases, pool = 2	0.313 (0.152–0.473)	0.156 (0.067–0.245)	0.171 (0.089–0.281)
All diseases, pool = 10	0.615 (0.532–0.699)	0.062 (0.048–0.075)	0.091 (0.073–0.112)
Low prevalence, unequal pools	0.008 (0.001–0.016) *	0.002 (0.000–0.004) *	0.002 (0.001–0.004)
Low prevalence, pool = 2 ^a^	0.063 (0.000–0.146)	0.031 (0.000–0.074)	0.032 (0.005–0.095)
Low prevalence, pool = 10	0.008 (0.000–0.023)	0.001 (0.000–0.002)	0.001 (0.000–0.003)
High prevalence, unequal pools	0.166 (0.136–0.196) *	0.037 (0.030–0.044) *	0.042 (0.035–0.051)
High prevalence, pool = 2	0.125 (0.010–0.240)	0.063 (0.003–0.122)	0.065 (0.021–0.144)
High prevalence, pool = 10	0.315 (0.236–0.395)	0.032 (0.022–0.041)	0.037 (0.027–0.050)

* Estimates disregard differences in pool size. ^a^ Data refers to *B. afzelii* instead of *B. garinii* for a lack of positive pools in the latter.

## Data Availability

The authors declare that data are available upon request to the corresponding author.
